# An informatic pipeline for the data capture and submission of quantitative proteomic data using iTRAQ^TM^

**DOI:** 10.1186/1477-5956-5-4

**Published:** 2007-02-01

**Authors:** Jennifer A Siepen, Neil Swainston, Andrew R Jones, Sarah R Hart, Henning Hermjakob, Philip Jones, Simon J Hubbard

**Affiliations:** 1 Faculty of Life Sciences, University of Manchester, M13 9PT, UK; 2 Manchester Interdisciplinary Biocentre, University of Manchester, UK; 3 School of Computer Science, Faculty of Engineering and Physical Sciences, University of Manchester, UK; 4 MBCMS, School of Chemistry, Manchester Interdisciplinary Biocentre, University of Manchester, UK; 5 EMBL Outstation EBI, Wellcome Trust Genome Campus, Hinxton, Cambs, UK

## Abstract

**Background:**

Proteomics continues to play a critical role in post-genomic science as continued advances in mass spectrometry and analytical chemistry support the separation and identification of increasing numbers of peptides and proteins from their characteristic mass spectra. In order to facilitate the sharing of this data, various standard formats have been, and continue to be, developed. Still not fully mature however, these are not yet able to cope with the increasing number of quantitative proteomic technologies that are being developed.

**Results:**

We propose an extension to the PRIDE and mzData XML schema to accommodate the concept of multiple samples per experiment, and in addition, capture the intensities of the iTRAQ^TM ^reporter ions in the entry. A simple Java-client has been developed to capture and convert the raw data from common spectral file formats, which also uses a third-party open source tool for the generation of iTRAQ^TM^ reported intensities from Mascot output, into a valid PRIDE XML entry.

**Conclusion:**

We describe an extension to the PRIDE and mzData schemas to enable the capture of quantitative data. Currently this is limited to iTRAQ^TM^ data but is readily extensible for other quantitative proteomic technologies. Furthermore, a software tool has been developed which enables conversion from various mass spectrum file formats and corresponding Mascot peptide identifications to PRIDE formatted XML. The tool represents a simple approach to preparing quantitative and qualitative data for submission to repositories such as PRIDE, which is necessary to facilitate data deposition and sharing in public domain database. The software is freely available from .

## Background

Proteomics continues to play a critical role in post-genomic science as continued advances in mass spectrometry and analytical chemistry support the separation and identification of increasing numbers of peptides and proteins from their characteristic mass spectra. A desirable trait for such a functional genomics technique is the ability to produce data on a genome-wide basis and, importantly, to be able to do this in a quantitative manner. In proteomics this means being able to quantify the protein changes in different conditions, be they temporal, pathogenic or environmental. Proteomics is beginning to address both these issues; wider genome coverage and quantitation of the proteins present. The latter has been driven by the continued development of techniques for the relative and absolute quantification of protein levels [1-6]. Equally, superior instrumentation and analytical approaches have improved the coverage of genomes, so that genome-wide quantitative proteomics is becoming a reality. This is epitomised by a recent quantitative study acquiring data for the majority of the yeast proteome [7], where the majority of proteins had peptide identifications with available quantitative data obtained using stable isotope labelling in cell culture (SILAC).

Clearly, these types of experiments will become more widespread and detailed. This presents several challenges to the proteomics community and the bioinformatics teams in particular, since it is desirable that this data is captured and stored in appropriate databases in consistent formats, to support data sharing and comparison. Although there are a growing number of data standards [8-12] and databases [13-18] for the storage of proteomic data, at present there is no formal model for quantitative proteomic data that has been fully developed. The Proteome Standards Initiative (PSI) and leading proteomics groups have helped drive the development of several standards for the mass spectral data itself, namely mzXML [8] and mzData [19], and these two are expected to soon merge. These support a comprehensive data model for the storage of proteomic-related mass spectral data, ranging from basic details about the sample, through instrument details and data processing steps, to the actual spectral lists of mass-to-charge values and intensities. This provides a relatively simple yet extensible format for any type of peptide or protein spectra, allowing users to support parent/precursor ion concepts and sophisticated MS^n ^experiments. Both formats utilise base64 encoding to represent the floating point mass-to-charge (m/z) and ion intensity pairs which form the core of the spectral information. Although this supports the capture of any protein, peptide or fragment ion MS spectra, quantitative data is not explicitly represented in the model. Furthermore, it is not clear how to link the spectra to rich descriptions of the experimental sample, or mixture of samples, within these formats. Indeed, the work to bring this together into a considered whole for proteomics and indeed, in a wider functional genomics context, is well underway, with standards in development for identifications (analysisXML), gels (GelML etc), general sample processing (spML) and functional genomics experiments more generally (FuGE, [11]). Even though the standards development community has not finished this process, database developers in proteomics have already provided solutions for many of these issues in the growing range of proteomics databases now available. These include PeptideAtlas [14], Open Proteomics Database (OPD) [13], Global Proteome Machine (GPM) [15], Pedro [17], PepSeeker [16], and the PRoteomics IDEntifications database (PRIDE) [18,20] as well as others. PeptideAtlas, GPM and PRIDE in particular already contain extensive collections of many millions of peptide identifications. PRIDE, for example, has integrated the mzData data standard into its own PRIDE XML format, which allows users to provide a rich description of their experiment and uses a range of well-supported ontologies to populate the model for a range of meta-data including taxonomy, instrument type, etc. The other databases are also able to capture a similar range of data.

At Manchester, local proteomics groups are active in quantitative proteomics, developing both novel methodology [5,6] and using existing technology to explore quantitative protein levels. In particular, the iTRAQ^TM^ technology [4] is widely used by many groups worldwide, since it offers several advantages, including the ability to multiplex several samples in one single experiment, quantifying several samples in one experiment via a series of reporter ions which are fragmented from an isobaric tag attached to free peptide amines. Thus, researchers can quantify the relative levels of several samples, averaging over data from several peptides, using a labelling technique applicable to all peptides, and not relying on cell culture or similar using stable isotope labelling. This ingenious technique presents informatics with a novel modelling challenge, since such a concept cannot be directly modelled in the existing mzData schema, which considers the sample itself to be a single entity to which all spectra in the experiment are related.

To address this problem, we have conducted a case study to further develop the PRIDE and mzData XML schema to accommodate the concept of multiple samples per experiment, and in addition capture the intensities of the iTRAQ^TM^ reporter ions in the entry. The model extensions are completely compatible with both the PRIDE and mzData schema, utilising controlled vocabulary terms which are added to the respective ontologies. Furthermore, we have developed a simple Java-client (the "Pride Wizard") to capture and convert the raw data from common spectral file formats, which also uses a third-party open source tool for the generation of iTRAQ^TM^ reported intensities from Mascot output. Together, this allows the user to capture large, high-throughput ITRAQ^TM^-based studies, without extensive repetitive manual data entry of individual peptide identifications, and delivered in a valid PSI-consistent data format (PRIDE XML) for submission to the PRIDE repository. The underlying model and Java-client are readily extended to other quantitation techniques. Finally, the Java-client also allows users to directly capture non-quantitative large scale proteomics data, providing the opportunity to convert Mascot-based spectral searches into mzData with associated peptide identifications. We believe this tool will allow proteomics groups to rapidly capture their datasets for submission to a PSI-sanctioned repository and provides a step change in the ease of complex proteomic data available for analysis and sharing for the community in general.

## Results

### Data capture pipeline

**Figure 1:**
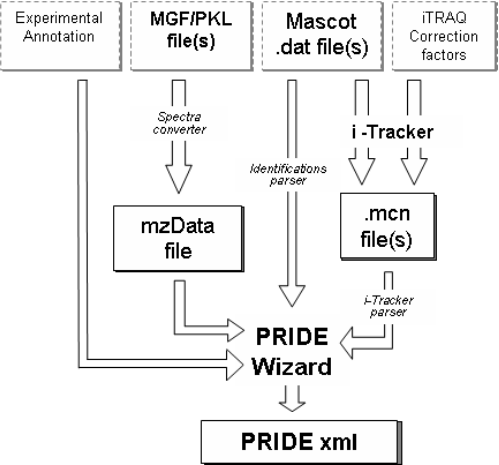
The data processing pipeline for the PRIDE wizard. Grey boxes represent the files/text that are required as input and the black boxes those files created by the PRIDE wizard.

The capture of the mass spectrometry data, associated protein and peptide identifications and quantitative values for multiple samples has been integrated into a single client application, shown in overview in Figure 1. In this example, the mass spectrometry data is represented in Mascot's .mgf format.

As input, the user provides the Pride Wizard with one or more processed mass spectrum files (in either .mgf, mzXML, .pkl or mzData format) and associated Mascot dat files containing protein and peptide identifications. In addition, a number of experimental meta data values are required.

**Figure 2:**
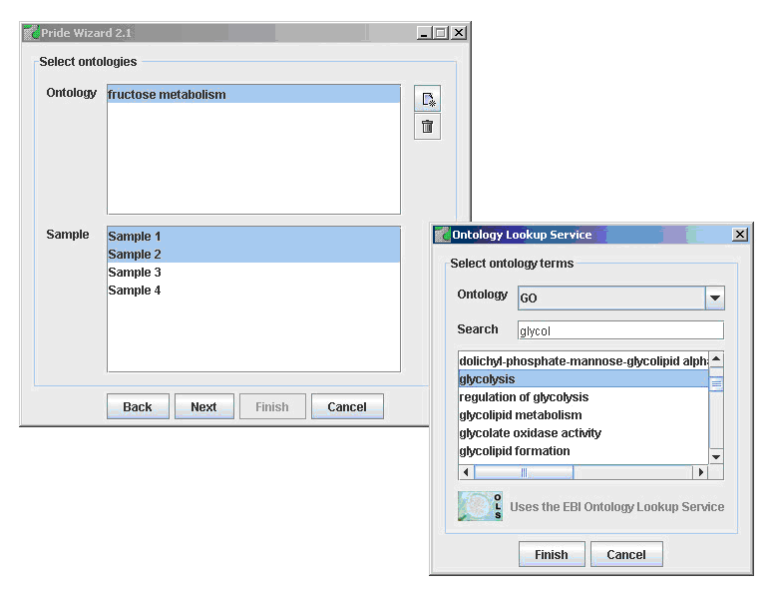
Ontology term selection in the PRIDE Wizard.

The Pride Wizard can be run in qualitative and quantitative iTRAQ^TM^ mode. In the case of the latter, the user specifies a number of samples involved in the experiment, and assigns one or more iTRAQ^TM^ labels to each of these samples. Ontology terms can also be assigned to the samples, as the Pride Wizard acts as a client to the EBI Ontology Lookup Service [21] (see Figure 2). A correction factor file must be submitted, in which the isotopic purity of each of the iTRAQ^TM^ reagents used in the experiment are specified.

Finally, the user specifies the location of the PRIDE XML file that will be generated upon successful completion of the wizard.

The conversion of this data into valid PRIDE XML takes a number of steps (shown in Figure 1). mzData is required in the final PRIDE XML document, so if necessary, submitted mass spectrum files are converted to mzData using a module provided by ProteomeCommons.org [22]. The ProteomeCommons module is again used to perform conversion of mzData to .mgf files, which are required by the identifications parser module.

The identifications parser takes the form of a Perl script which parses an .mgf mass spectrum file and a Mascot 'dat' file to generate a PRIDE XML fragment containing protein and peptide identifications. In the case where the user has provided iTRAQ^TM^ labelled sample data the peptide identification results from the Mascot 'dat' file are merged with iTRAQ^TM^ intensities and ratios from i-Tracker [23].

Only the top three ranking identified peptides are reported in the PRIDE XML and the identified peptides are grouped according to the protein accession for the first matching protein for each of the identifications. Where a post-translational modification is assigned by Mascot (fixed or variable) then the name of the modification is matched to the UNIMOD database [24]. If the name of the modification cannot be matched to UNIMOD then the name of the peptide modification is represented as a userParam (see Methods for a description of the schema constructs used).

The i-Tracker software returns the relative ratios of each of the iTRAQ^TM^ reporter ions from an .mgf formatted file, a set of correction factors and a user-defined threshold. If the maximum ion peak intensity for any reporter ion peak area is equal to or less than the user-entered threshold a flag of "UT" for "Under Threshold" is reported in the PRIDE XML file.

The iTRAQ^TM^ intensities are reported using the iTRAQ^TM^ reagent 114 label (see Methods). The actual ratios for each of the iTRAQ^TM^ reporter ions, calculated by i-Tracker, are represented as userParams, where, for every peptide identification, we represent the iTRAQ^TM^ reporter ion ratios as:

<userParam value="1" name="114_114"/>

<userParam value="1.597" name="114_115"/>

...

<userParam value="1.233" name="117_116"/>

<userParam value="1" name="117_117"/>

The final step involves merging together each of the mzData files with the generated PRIDE XML fragments to generate a single PRIDE XML document representing the entire experiment. This document is then saved to the user-specified location.

### Test data

The software tool was tested on selection of exemplar quantitative data from a number of different collaborating laboratories and successfully created valid PRIDE XML files. The samples included iTRAQ^TM^-based analyses from multiple species, using several instrument types. Full details of the experiments are contained in the methods. The performance of the software was estimated; the wizard takes approximately 4.3 minutes to run on 2314 .mgf formatted mass spectra with 3581 corresponding peptide identifications on a single laptop.

## Discussion

We have described a use of controlled vocabulary terms to represent quantitative proteomics data within the PRIDE data format and a software tool to capture and produce the correct file format. Several data standards are currently under development by the Proteomics Standards Initiative which will be adopted by PRIDE, allowing a complete proteomics pipeline to be represented. This includes detailed descriptions of protein or peptide separations and labelling (in spML), the mass spectrometry data (mzData) and the protein identifications and quantitative values (analysisXML). However, it is unlikely that spML and analysisXML will be stable and implemented by PRIDE until late 2007 or early 2008. Therefore, the format extension proposed here represents a suitable interim solution for storing quantitative data, and we encourage other laboratories to adopt the conventions. This will allow quantitative data to be represented now in a "pseudo-standard" format and will enable other groups to download such data from PRIDE and perform re-analysis.

In addition to this functionality for iTRAQ^TM^ based data, we believe the tool is readily extensible for other quantitative proteomic technologies in a similar fashion, by extending the model and making minor adaptations to the associated Perl and Java code in the Pride Wizard. To this end, we have made the source code available [25]. As data capture needs for both SILAC [1] and QconCAT [5,6] methodologies are underway in our laboratories we expect to provide specific solutions for these approaches in early 2007.

Although the tool was designed ostensibly solely for quantitative data capture, it clearly is able to capture large volumes of identification data and deliver this automatically in PRIDE XML format. We anticipate this will be extremely useful to many groups with high-throughput data sets they wish to capture without tedious manual input. In order to capture the associated experiment, instrument and sample data that can be associated with a PRIDE entry we recommend the PedroDC data capture tool developed at Manchester [26]. Since the PRIDE XML delivered by our pipeline validates against the PRIDE schema, the data capture tool allows further flexibility to load the PRIDE XML and make suitable additions and edits. Alternatively, the PRIDE team have developed a spreadsheet-based approach linked directly to the Ontology Lookup Service at the EBI which provides an efficient means of entering the higher level data into a PRIDE entry. We anticipate that all of the above will be useful to different user groups, and that a suite of different approaches are probably necessary in proteomics, as any enhancement of data capture capabilities which facilitates data deposition and sharing in public domain repositories is to be welcomed.

## Methods

### Data capture overview

To generate iTRAQ^TM^ quantitative data requires several key component data types which must be integrated. An overview of these data types and the associated analysis tools are shown in Figure 1. A typical iTRAQ^TM^ experiment involves the analysis of several samples in a single MS run where peptides are identified in a standard fashion using a search tool such as Mascot [27]. The spectral data are typically delivered to Mascot using Matrix Science's Mascot Generic Format (.mgf), although the tool can cope with a variety of vendor specific formats, as well as mzData. The peptide identifications themselves are contained in Mascot's .dat output file. Finally, to generate the quantitative data for each peptide, users can employ ABI's ProQuant software, or if they prefer, third-party open source tools such as i-Tracker [23]. The latter uses a correction file supplied by ABI to adjust the reporter ion intensities for each identified peptide. The Pride Wizard we have developed integrates these data into a single PRIDE XML file. The model extensions are detailed in the following section.

### Modelling quantitative data in PRIDE XML

The mzData schema lies at the heart of a PRIDE XML entry. PRIDE's model is deliberately "light touch" whilst data standards mature, and is readily extensible via inbuilt controlled vocabulary (CV) terms. However, mzData contains only a single sample description object which is also used by PRIDE to capture sample information.

**Figure 3:**
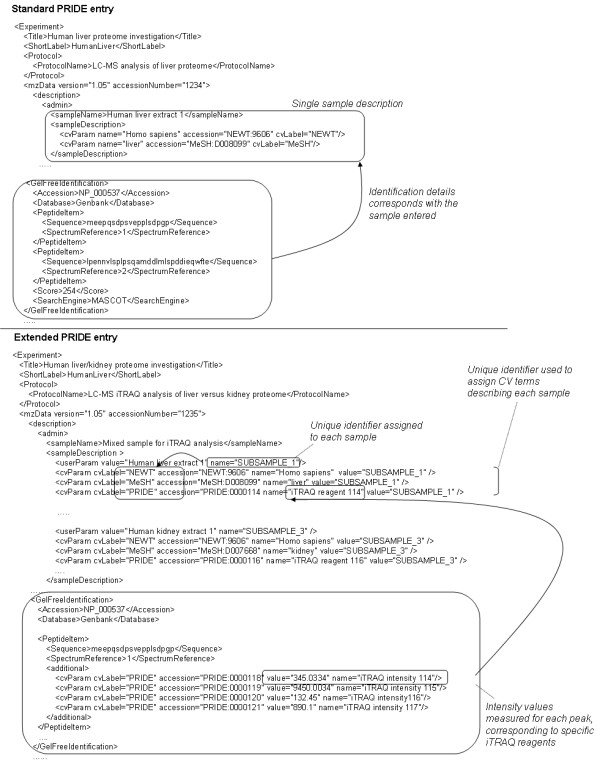
Extensions to the PRIDE XML schema.

Controlled vocabularies are frequently employed in data formats to provide a consistent extension mechanism allowing a format to capture unanticipated data types [12]. PRIDE files can be annotated with CV and user-defined terms to describe details of the experimental protocol employed, the sample analysed, the instrument used and protein or peptide identifications. We have made use of CV and user-defined terms in PRIDE to support multiplexed sample descriptions and the corresponding quantitative data for each sample (Figure 3)

A CV term in PRIDE has a name (the term itself), a unique accession from the source CV, a label to identify the CV source and optionally a value that can be completed by the user. An example is as follows, where the term *Homo sapiens *(from the NEWT taxonomy) [28] is used to describe the sample:

<sampleDescription>

<cvParam name="Homo sapiens" accession="NEWT:9606" cvLabel="NEWT"/>

</sampleDescription>

A further example, where a user-entered value (2000 for "Mass Resolution") has been included:

<analyzer>

<cvParam accession="PSI:1000011" name="Mass Resolution" value="2000" cvLabel="PSI"/>

</analyzer>

In the extension of PRIDE, we have utilised the userParam facility and the CV representation to capture the names of multiple samples within a single file. The userParam term supports the inclusion of a placeholder for the name of each sub-sample (SUBSAMPLE_1 is used in the example below). This is easily supplemented by the use of additional CV terms in the standard way to add taxonomic or further related information pertaining to the sub-sample. Finally, we have created a further list of CV terms named after the 4 standard iTRAQ^TM^ report ions to link the sub-samples to specific reagents called iTRAQ^TM^ reagent 114, iTRAQ^TM^ reagent 115 etc. The user completes the value attribute of cvParam with the name of each sample they wish to record in the file.

<userParam value="Human Liver Extract 1" name="SUBSAMPLE_1"/>

<cvParam cvLabel="PRIDE" accession="PRIDE:0000114" value="SUBSAMPLE_1" name="iTRAQ reagent 114">

The term SUBSAMPLE_1 then serves as a unique identifier for that sample throughout the rest of the file. Where the user wants to add further CV terms to describe the sample, the value attribute is completed with SUBSAMPLE_1.

<sampleDescription>

<cvParam name="Homo sapiens" accession="NEWT:9606" cvLabel="NEWT" value="SUBSAMPLE_1"/>

</sampleDescription>

In order to enter the actual intensities of the reporter ions, we propose the following convention, adapting the iTRAQ^TM^ reagent 114 label further to iTRAQ^TM^ intensity 114 as shown below.

<cvParam cvLabel="PRIDE" accession="PRIDE:0000118" value="0.048" name="iTRAQ intensity 114"/>

<cvParam cvLabel="PRIDE" accession="PRIDE:0000119" value="0.193" name="iTRAQ intensity 115"/>

<cvParam cvLabel="PRIDE" accession="PRIDE:0000120" value="0.204" name="iTRAQ intensity 116"/>

<cvParam cvLabel="PRIDE" accession="PRIDE:0000121" value="0.65" name="iTRAQ intensity 117"/>

The terms proposed here have been added to the PRIDE CV and have been assigned stable accession numbers. It is apparent from this example that other quantitative data with intensity or ratio values, calculated in a variety of ways, can be represented using similar CV terms.

### Test data

The software tool was tested on a number of different data sets from different laboratories. Test set 1 was derived from *Trypanosoma brucei *flagellum samples which were prepared as described previously [29]. Samples were derivatised using iTRAQ^TM^ according to the manufacturer's instructions and derivatised peptides from four samples were prepared and analysed online with a QTOF I instrument (Waters, Manchester, upgraded to QTOF II specifications by MS Horizons, Manchester). Data acquisition was performed using MassLynx 3.4, acquiring 3 channels of tandem MS data. Following acquisition, data were processed using ProteinLynx to generate .pkl files.

Test set 2 was derived from soluble extracts from the gram negative plant pathogenic bacterium *Erwinia carotovora *(sp atroseptica SCRI1043) which were prepared as described previously [30]. Three biological replicate samples were labelled with iTRAQ^TM^ reagents 114–116 respectively, a fourth sample which consisted of a pool of the three replicates was labelled with the 117 iTRAQ^TM^ reagent. Labelling, multidimensional LC and MSMS were carried out as in [31]. The data submitted to the PRIDE wizard was essentially from the combination of running four fractions from strong cation exchange column on LCMSMS (QSTAR, Applied Biosystems).

Test set 3 was derived from primitive hematopoietic cells from mouse bone marrow as described previously [32]. Samples were derivatised using iTRAQ^TM^ according to the manufacturer's instructions and derivatised peptides from four samples were prepared and analysed online with a QSTAR XL (Applied Biosystems). Data acquisition was performed using an independent data acquisition protocol as described previously [32].

## Availability and requirements

Project name: Pride Wizard

Project homepage: 

Operating system: Windows

Programming language: perl, Java 1.4.2 and above.

Licence: GNU GPL

## Authors' contributions

SJH, ARJ, JAS, NS & SRH designed and developed the quantitative data model in the PRIDE XML schema, and jointly proposed the schema extensions with subsequent additional verification from PJ & HH. JAS & NS developed the software tool to capture the data, with testing from SRH. SJH conceived the study, lead the manuscript production with contributions from all authors, who have read and approved the final manuscript.

## Declaration of competing interests

The author(s) declare that they have no competing interests.
